# Percutaneous endovascular treatment of infrainguinal PAOD: Results of the PSI register study in 74 German vascular centers

**DOI:** 10.1007/s00772-016-0202-2

**Published:** 2016-10-28

**Authors:** C.-A. Behrendt, F. Heidemann, K. Haustein, R. T. Grundmann, E. S. Debus, PSI Collaborators, K. M. Balzer, R. Banafsche, L. Barbera, M. Baumhäkel, E. Blajan, H. Böhner, P. Breuer, U. Brune, J. Brunkwall, T. Bürger, S. Classen, A. Cöster, P. Dahl, E. S. Dus, J. P. Delgado, H. Dill, S. Eder, T. Fährenkemper, B. Feidicker, J. Forkel, J. Gahlen, B. Geier, R. Ghoi, H. Görtz, J. Gräbedünkel, W. Gunkel, O. Hader, T. Hammermüller, J. Hatzl, J. Hoffmann, M. Hofmann, U. Huberts, M. Jacobs, V. Kiechle, M. Kindermann, M. Kleemann, P. Kolka, M. Krahl, T. Krönert, G. Krupski-Berdien, H. Kuffner, M. Kuhnert, T. Lange, V. K. Lauff, T. Lesser, F. Liewald, D. Lommel, M. Naundorf, K. Nitschmann, S. Nöldeke, T. Noppeney, A. Oberhuber, D. M. Ockert, K.-H. Orend, U. Ossig, C. Petridis, A. Pflugradt, U. Quellmalz, P. Reimer, J. Remig, P. Richter, G. Riepe, G. Rümenapf, T. Schaefer, H. Schelzig, G. Schmidt, M. Schneider, J. Schofer, S. Seifert, M. Siggelkow, S. Sixt, E. Stautner, A. Stehr, M. Storck, J. Teßarek, O. E. Teebken, K.-D. Thom, W. P. Tigges, G. Voshage, K.-D. Wagenbreth, K. P. Walluscheck, D. Walter, R. Weidenhagen, B. Weis-Müller, H. Wenk, M. Wenk, M. Wiedner, J. Wilde, F. T. Wittstock

**Affiliations:** 1 Department of Vascular Medicine, University Heart Center Hamburg, University Medical Center Hamburg-Eppendorf, Hamburg, Germany; 2 German Institute of Vascular Medicine and Health Research (DIGG) of the DGG, Berlin, Germany

**Keywords:** Percutaneous infrainguinal stent, Peripheral arterial occlusive disease, Endovascular, Percutaneous, GermanVasc, PSI, PAVK, Endovaskulär, Perkutan, GermanVasc

## Abstract

**Background:**

The percutaneous infrainguinal stent (PSI) register study aimed to collate all percutaneous endovascular procedures for infrainguinal peripheral arterial occlusive disease (PAOD) conducted in 74 German vascular centers between September and November 2015 (3 months). In order to obtain representative results all consecutive treatment procedures had to be submitted by the participating trial centers.

**Material and methods:**

This was a prospective, nonrandomized multicenter study design. All patients suffering from intermittent claudication (IC, Fontaine stage II) or critical limb ischemia (CLI, Fontaine stages III and IV) were included. Trial centers with less than 5 cases reported within the 3‑month trial period or centers that could not ensure the submission of all treated patients were excluded.

**Results:**

In the final assessment 2798 treated cases from 74 trial centers were reported of which 65 (87.8 %) centers were under the leadership of a vascular surgeon. Approximately 33 % of the interventions in centers under the leadership of vascular surgeons were conducted by radiologists. Risk factors, especially chronic renal disease, diabetes and cardiac risk factors were significantly different between patients with IC and CLI. Of the patients with Fontaine stage II PAOD 41.3 % had 3 patent crural vessels compared to only 10.8 % of patients with Fontaine stage IV. With respect to peri-interventional complications, percutaneous endovascular treatment of IC was a safe procedure with severe complications in less than 1 % and no fatalities. Only 4.5 % of the procedures were conducted under ambulatory conditions. In the supragenual region self-expanding bare metal stents, standard percutaneous transluminal angioplasty (PTA) and drug-coated balloons were the most frequently used procedures. For interventions below the knee, standard PTA was the most commonly employed treatment.

**Conclusion:**

The main aim of the PSI study was to obtain a realistic picture of percutaneous endovascular techniques used to treat suprapopliteal and infrapopliteal PAOD lesions and to describe the treatment procedures used by vascular specialists in Germany. To investigate the change in trends for treatment over time, this study has to be repeated in the future in order to test how quickly the results of randomized studies can be implemented in practice.

****Electronic supplementary material**:**

A complete list of the PSI study collaborators is available under doi: 10.1007/s00772-016-0202-2.

## Zusammenfassung

**Hintergrund:**

Die „Percutaneous-infrainguinal-stent“(PSI)-Register-Studie sollte über einen Zeitraum von 3 Monaten an einer möglichst repräsentativen Zahl von mehrheitlich gefäßchirurgischen Gefäßzentren konsekutiv alle perkutanen endovaskulären Behandlungen bei infrainguinaler peripherer arterieller Verschlusskrankheit (PAVK) erfassen und dadurch einen Einblick in die Versorgungsrealität ermöglichen.

**Material und Methode:**

Es handelte sich um eine prospektive, nichtrandomisierte Multizenterstudie. Eingeschlossen wurden Patienten mit einer intermittierenden Claudicatio (IC, Fontaine-Stadium II) und einer kritischen Extremitätenischämie (CLI, Fontaine-Stadien III und IV). Zentren mit einer Datenübermittlung von weniger als 5 Behandlungsfällen in 3 Monaten oder nichtbestätigter konsekutiver Datenübermittlung wurden von der Teilnahme ausgeschlossen.

**Ergebnisse:**

In die Endauswertung gingen insgesamt 2798 Behandlungsfälle aus 74 Studienzentren ein. Von den 74 Zentren standen 65 (87,8 %) unter gefäßchirurgischer Leitung. Etwa 33 % der Interventionen in gefäßchirurgisch geführten Zentren wurden durch Radiologen durchgeführt. Patienten mit IC und CLI unterschieden sich signifikant in ihren Risikofaktoren, wobei besonders chronische Nierenerkrankungen, Diabetes mellitus und kardiale Risikofaktoren zu betonen sind. Es wiesen 41,3 % der Patienten mit PAVK im Fontaine-Stadium IIi 3 offene Unterschenkelgefäße auf, verglichen mit lediglich 10,8 % der Patienten im Fontaine-Stadium IV. Die perkutane endovaskuläre Behandlung der IC war hinsichtlich schwerer periinterventioneller Komplikationen ein sicheres Verfahren, schwere Komplikationen traten in weniger als 1 % auf, keine Todesfälle bei IC. Nur 4,5 % aller Behandlungen wurden unter ambulanten Bedingungen durchgeführt. In der suprapoplitealen Strombahn wurde der selbstexpandierende „bare metal stent“ vor dem Standard-verfahren der perkutanen transluminalen Angioplastie (PTA) und dem medikamentenbeschichteten Ballon am häufigsten verwendet. In der infrapoplitealen Strombahn fand die Standard-PTA den häufigsten Zuspruch.

**Schlussfolgerung:**

Wesentliche Aufgabe der PSI-Register-Studie war es, die angewandten endovaskulären Techniken bei supra- und infrapoplitealen Läsionen zu erfassen sowie Daten für eine Beschreibung der Istsituation zu erheben. Dies soll es in Zukunft – bei Wiederholung der Untersuchung – ermöglichen, Behandlungstrends nachzuweisen, um überprüfen zu können, wie schnell sich die Ergebnisse (randomisierter) Studien in der Praxis umsetzen lassen.

## Introduction

This study was concerned with the endovascular treatment of peripheral arterial occlusive disease (PAOD) and the techniques employed. Numerous guidelines on this topic have already been published. The version of the S3 guidelines published in 2015 on the diagnostics, therapy and follow-up care of PAOD by the German Society for Angiology – Society for Vascular Medicine [1], suggests that when considering endovascular treatment of medium to large size femoropopliteal lesions, the primary stent angioplasty with nitinol stents is the preferred choice over balloon angioplasty with secondary stent implants (bail out stents). Conversely, when considering treatment of infrapopliteal vessel lesions, a stent implant should only be considered when balloon percutaneous transluminal angioplasty (PTA) does not produce satisfactory angiographic results. Although the value of the new drug-eluting balloons in the angioplasty of infrapopliteal arteries could not be adequately assessed in the guidelines, drug-eluting (paclitaxel-coated) balloons were, rather vaguely, deemed useful in the endovascular treatment of femoropopliteal lesions, particularly when reduced risk of restenosis and reintervention are considered clinically essential (a goal that physicians in vascular medicine should always strive for). The new practice guidelines from the Society for Vascular Surgery (SVS) [2] recommend selective stenting of focal lesions of the superficial femoral artery if PTA alone produces unsatisfactory results (bail out) and nitinol stenting (with or without paclitaxel) for medium length lesions. The importance of new techniques in the clinical practice appears to be unclear, as shown in a consensus document from the Society for Cardiovascular Angiography and Interventions (SCAI) [3]. The document calls for additional data concerning the application of drug-eluting balloons, drug-eluting stents (DES), bioresorbable stents, cutting balloons, cryoplasty, laser, rotary and directional atherectomy, before wide propagation can begin particularly with regard to cost effectiveness.

The variety of the techniques listed here prompted us to raise the question of the frequency in which German vascular surgeons, radiologists and angiologists currently apply these methods in the endovascular treatment of infrainguinal PAOD. Here, a further distinction between femoropopliteal and infrapopliteal lesions was made. The current study is to serve as a form of inventory so that treatment trends and developments can be assessed following future repeat and follow-up investigations.

## Patients and methods

This was a prospective, non-randomized, multicentric study design. The percutaneous infrainguinal stent (PSI) registry study included all percutaneous endovascular treatment in patients with infrainguinal PAOD from 1 September 2015 to 30 November 2015 (91 days) across 79 participating German centers. A total of 200 centers (all levels of care) were asked to participate in the study, while vascular surgeons were the primary points of contact. If it was revealed that such procedures were not performed on the patient wards of the departments, other neighboring departments were contacted. Willingness to participate in the study was 40 %. Patients with intermittent claudication (IC, Fontaine stage II) or critical limb ischemia (CLI, Fontaine stages III or IV) were included. Each intervention performed was documented as an individual treatment case, so that patients could be included multiple times if interventions were repeated. Data collection and anonymous submission to the trial center were carried out by the participating centers. Trial centers with less than 5 treatment cases reported within the 3‑month trial period or centers that could not ensure the submission of all treated patients were excluded from participation. In total, 10 reported treatment cases were excluded.

Based on the fact that the PSI study data consisted of anonymized treatment data from routine operations, which did not allow patient identification, the medical council ethics committees of both Hamburg and Düsseldorf found that the study was not within their consulting jurisdiction and therefore posed no objections to the planned studies. The statistical analysis of the data was performed with SPSS version 22.0 (Armonk, NY). A group comparison with respect to the metric variables was done with Student’s t‑test (for normally distributed variables) or with the Mann-Whitney U‑test (for non-normally distributed variables). For categorical variables, Fisher’s exact test or the χ^2^-test was employed. The significance level was chosen to be *p* < 0.05. Because it was not possible to perform a plausibility check for data in paper form and because obligatory data were not always reported in their entirety, only valid data (valid percentages) were used in the calculations. As a result, the population size differed among the selected variables (Table 1). This has been noted in the individual collectives.

**Table 1:** Reported variables for endovascular therapy of patients with infrainguinal peripheral arterial occlusive disease

Variable	Reported data	%
Clinic identifier (cases)	2798	100
Treated side (left or right)	2763	98.7
Sex	2761	98.7
Specialist discipline	2626	93.9
Treated artery/flow path	2701	96.5
Indication for treatment	2754	98.4
Outflow prior to intervention	2707	96.7
Closure system	2774	99.1
Outflow following intervention	2763	98.7
Result	2727	97.5
Discharge goal(s)	2716	97.1

## Results

### Participating centers and specialist disciplines

**Fig. 1:**
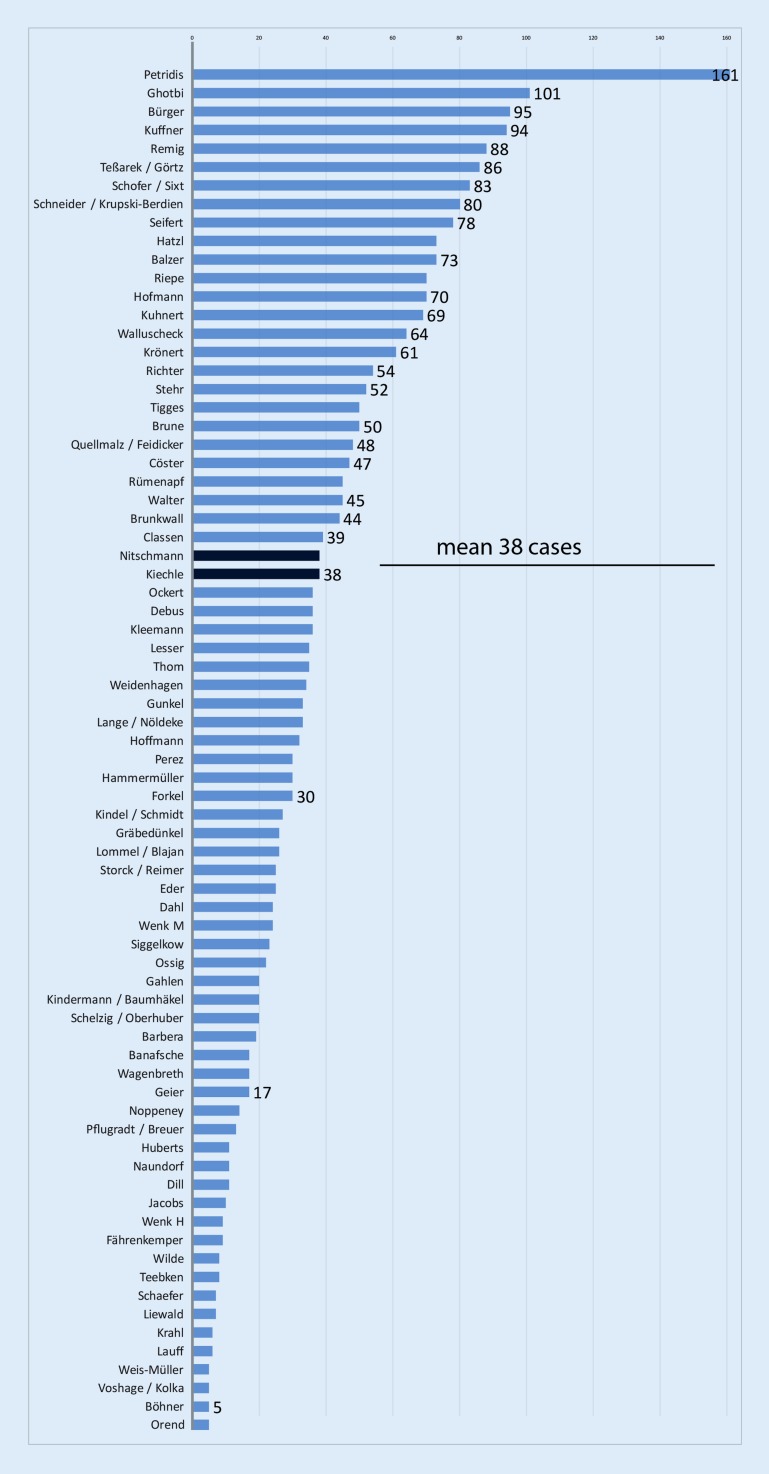
Reported data from the participating trial centers as name of the chief investigator(s) (average 38 cases per center)

A total of 79 centers were eligible for the PSI register study by committing to report data from all treatment cases. Cases from 5 centers could not be included as either the minimum of 5 cases in 3 months was not achieved or the complete submission of data could not be confirmed or validated. A total of 2798 cases received from 74 trial centers were included in the final analysis (Fig. 1). On average, 38 cases per center (range 5–161) were documented. According to the information on the website, 65 of the 74 participating centers (87.8 %) were headed by a vascular surgeon (ward with patient beds), the remainder were distributed among departments under mixed leadership (*n* = 2), radiology (*n* = 2) and angiology (*n* = 5). The primary discipline of medical personnel who carried out the interventions was indicated to be 1517 out of 2626 (57.8 %) in vascular surgery, 794 (30.2 %) in radiology and 307 (11.7 %) in angiology. On the patient wards in the vascular surgery departments 60.1 % of the interventions were performed by vascular surgeons themselves, 32.8 % by radiologists and 6.9 % by angiologists.

### Patient collective

In 2761 out of 2798 treatment cases, information regarding patient sex was provided. A total of 1696 (61.4 %) men and 1065 (38.6 %) women were enrolled in this study. The average age of the male patients was 70.4 years (SD 10.0, median 71 years). The treated women were with an average age of 75.8 years (SD 10.5, median 77 years) significantly older (*p* < 0.001). The frequency of treatment for the right and left lower limbs (52.2 % vs. 47.8 %, respectively) did not significantly differ. Out of 2704 treatment cases 1798 (66.5 %) were primary operations, whereas in 906 of the cases (33.5 %) patients had also been previously treated. Comorbidities and risk factors of patients, grouped according to gender are listed in Table 2. Almost 50 % of the men and 39.3 % of women also suffered from diabetes mellitus. Severe chronic renal failure stage 4 and 5 with a glomerular filtration rate (GFR) <30 ml/min was indicated in 11.2 % of men and 12.6 % of women and 4.4 % of men and 3.5 % of women were dialysis-dependent. Furthermore, there were significantly more active smokers among the men (30.8 % vs. 20.6 % of the women). Table 3 provides an overview of the risk factors in patients with IC and CLI. Patients with CLI suffered significantly more frequently from atrial fibrillation (22.8 % vs. 8.0 %, *p* < 0.001), diabetes mellitus (57.1 % vs. 31.8 %, *p* < 0.001) and chronic kidney disease (17.6 % vs. 5.0 %, *p* < 0.001) and were more frequently dialysis-dependent, when compared to patients with IC. In addition, patients suffering from CLI were on average 75 years of age and therefore significantly older than patients with IC (average 69.4 years).

**Table 2:** Distribution of risk factors of 2761 treatment cases separated according to sex

Risk factor	Men	Women	*p*-Value
	*n* = 1696	*n* = 1065	
Age (years, median)	70.39 (71)	75.81 (77)	<0.001
Active smoker status	522/1696 (30.8 %)	219/1065 (20.6 %)	<0.001
Coronary heart disease	660/1696 (38.9 %)	353/1065 (33.1 %)	0.002
Acute coronary syndrome (in the previous 6 months)	47/1696 (2.8 %)	18/1065 (1.7 %)	n. s.
Atrial fibrillation	255/1696 (15.0 %)	190/1065 (17.8 %)	n. s.
Obesity (BMI >30 kg/m^2^)	197/1696 (11.6 %)	140/1065 (13.1 %)	n. s.
Diabetes mellitus	838/1696 (49.4 %)	419/1065 (39.3 %)	<0.001
Chronic renal disease (stage 4/GFR <30 ml/min)	190/1696 (11.2 %)	134/1065 (12.6 %)	n. s.
Dialysis dependency	74/1696 (4.4 %)	37/1065 (3.5 %)	n. s.
Stroke or TIA (in the previous 6 months)	24/1696 (1.4 %)	10/1065 (0.9 %)	n. s.

*BMI* body mass index, *GFR* glomerular filtration rate, *TIA* transient ischemic attack, *n.s.* not significant

**Table 3:** Distribution of risk factors according to indications for treatment (data from 2754 cases)

Risk factor	Intermittent claudication	Critical limb ischemia	*p*-Value
	*n* = 1259	*n* = 1495	
Men *n* (%)	782/1240 (63.1 %)	885/1477 (59.9 %)	n. s.
Age (years, median)	69.43 (70)	75.00 (76)	<0.001
Active smoker status	482/1259 (38.3 %)	264/1495 (17.7 %)	<0.001
Coronary heart disease	409/1259 (32.5 %)	602/1495 (40.3 %)	<0.001
Acute coronary syndrome (in the previous 6 months)	25/1259 (2.0 %)	40/1495 (2.7 %)	n. s.
Atrial fibrillation	101/1259 (8.0 %)	341/1495 (22.8 %)	<0.001
Obesity (BMI >30 kg/m^2^)	136/1259 (10.8 %)	207/1495 (13.8 %)	0.016
Diabetes mellitus	400/1259 (31.8 %)	853/1495 (57.1 %)	<0.001
Chronic renal disease (stage 4 and 5/GFR <30 ml/min)	63/1259 (5.0 %)	263/1495 (17.6 %)	<0.001
Dialysis dependency	14/1259 (1.1 %)	96/1495 (6.4 %)	<0.001
Stroke or TIA (in the previous 6 months)	15/1259 (1.2 %)	18/1495 (1.2 %)	n. s.

*BMI* body mass index, *GFR* glomerular filtration rate, *TIA* transient ischemic attack, *n.s*. not significant

### Treatment indications and outflow prior to intervention

**Fig. 2:**
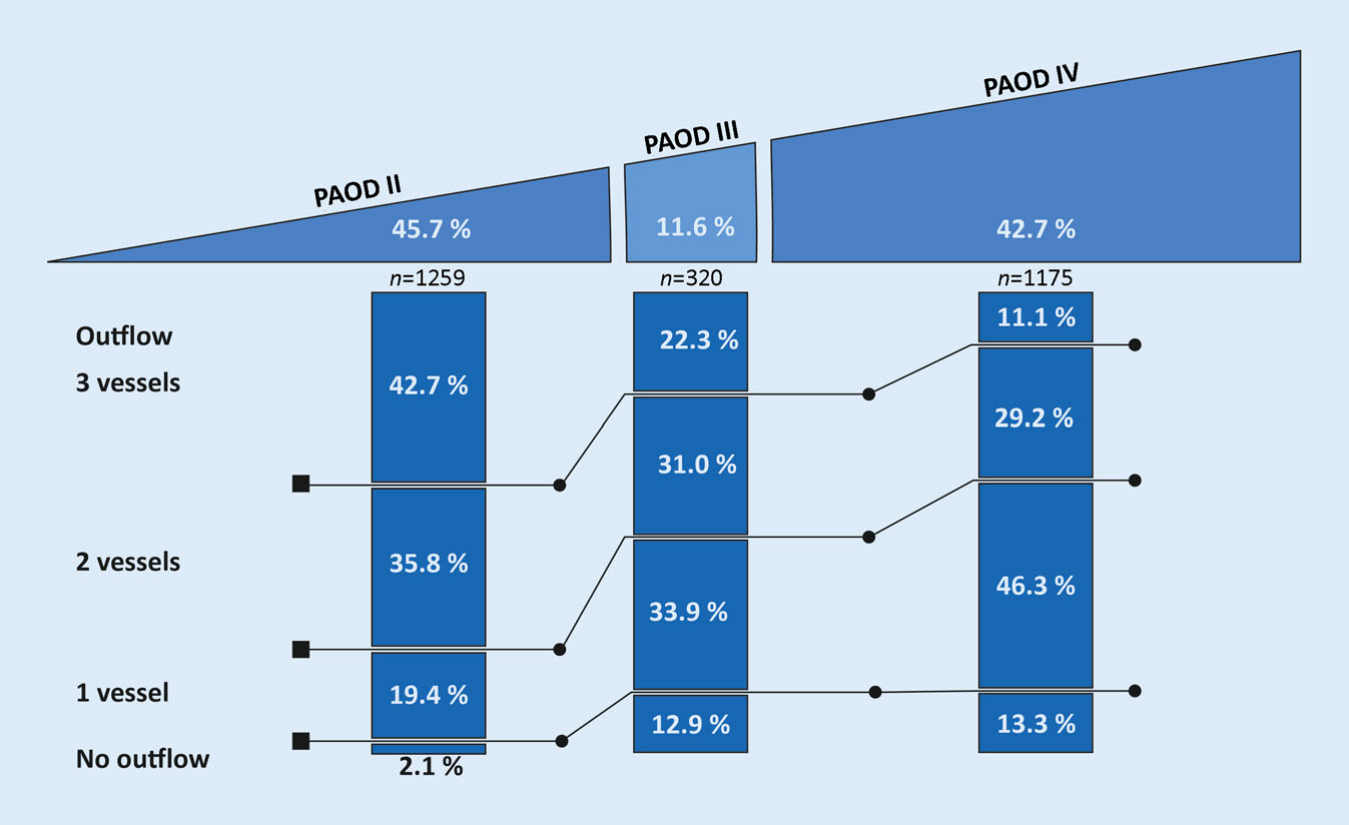
Distribution of treatment indications and outflow prior to intervention, grouped according to indications (*PAOD* peripheral arterial occlusive disease, Fontaine stage)

Treatment indications and outflow prior to intervention are listed in Fig. 2. The majority of the procedures carried out (1259 out of 2754 or 45.7 %) were due to PAOD stage II (intermittent claudication), followed by PAOD stage IV (1175 or 42.7 %) and stage III (320 or 11.6 %). Patency of 3 crural vessels was observed in 41.3 %, 21.6 % and 10.8 % of the stage II, III and IV treatment cases, respectively.

### Anesthesia

The operation was performed with the patient under local anesthesia in 2284 (83.0 %) of the cases.

### Procedural approach and closure

In 1796 (64.2 %) of the procedures access was achieved via inguinal antegrade puncture, while 889 (31.8 %) cases were performed using an inguinal retrograde approach (crossover). A brachial and popliteal vascular approach was performed in only 26 and 6 cases, respectively. Inguinal antegrade puncture was the most frequently selected access in both vascular surgical and radiological interventions (64.0 % and 82.6 %, respectively). Angiology interventions were preferred over the inguinal retrograde crossover approach, being performed in 61.1 % of the cases. When deciding which method was best to use in the treatment of vascular puncture site, simple compression bandages as well as a variety of common closure devices were also investigated. Compression bandages were most frequently applied in 43.6 % of cases. Second and third places were AngioSeal (24.9 %) and ExoSeal (11.8 %), respectively. The technique of vessel closure by FemoStop was almost exclusively employed by vascular surgeons. An overview of the distribution of the various methods for the treatment of vascular puncture site is provided in Table 4.

**Table 4:** Choice of closure system according to treating specialist discipline

	Total	Vascular surgery	Radiology	Angiology	Other
	*n* = 2774	*n* = 1499	*n* = 790	*n* = 305	*n* = 8
Compression Bandage	1210 (43.6 %)	583 (38.9 %)	452 (57.2 %)	101 (33.1 %)	2 (25 %)
AngioSeal (S. Jude Medical GmbH, Eschborn, Germany)	692 (24.9 %)	337 (22.5 %)	150 (19.0 %)	147 (48.2 %)	1 (12.5 %)
ExoSeal (Cordis, Baar, Switzerland)	328 (11.8 %)	121 (8.1 %)	157 (19.9 %)	33 (10.8 %)	0
StarClose (Abbott Vascular, Santa Clara, USA)	235 (8.5 %)	193 (12.9 %)	18 (2.3 %)	3 (1.0 %)	5 (62.5 %)
FemoStop (St. Jude Medical, Eschborn, Germany)	124 (4.5 %)	121 (8.1 %)	1 (0.1 %)	1 (0.3 %)	0
PerClose (Abbott Vascular, Santa Clara, USA)	10(0.4 %)	7(0.5 %)	3(0.4 %)	0	0
Other	175 (6.3 %)	137 (9.1 %)	9 (1.1 %)	20 (6.6 %)	0

### Treated vessel, procedures and devices

**Fig. 3:**
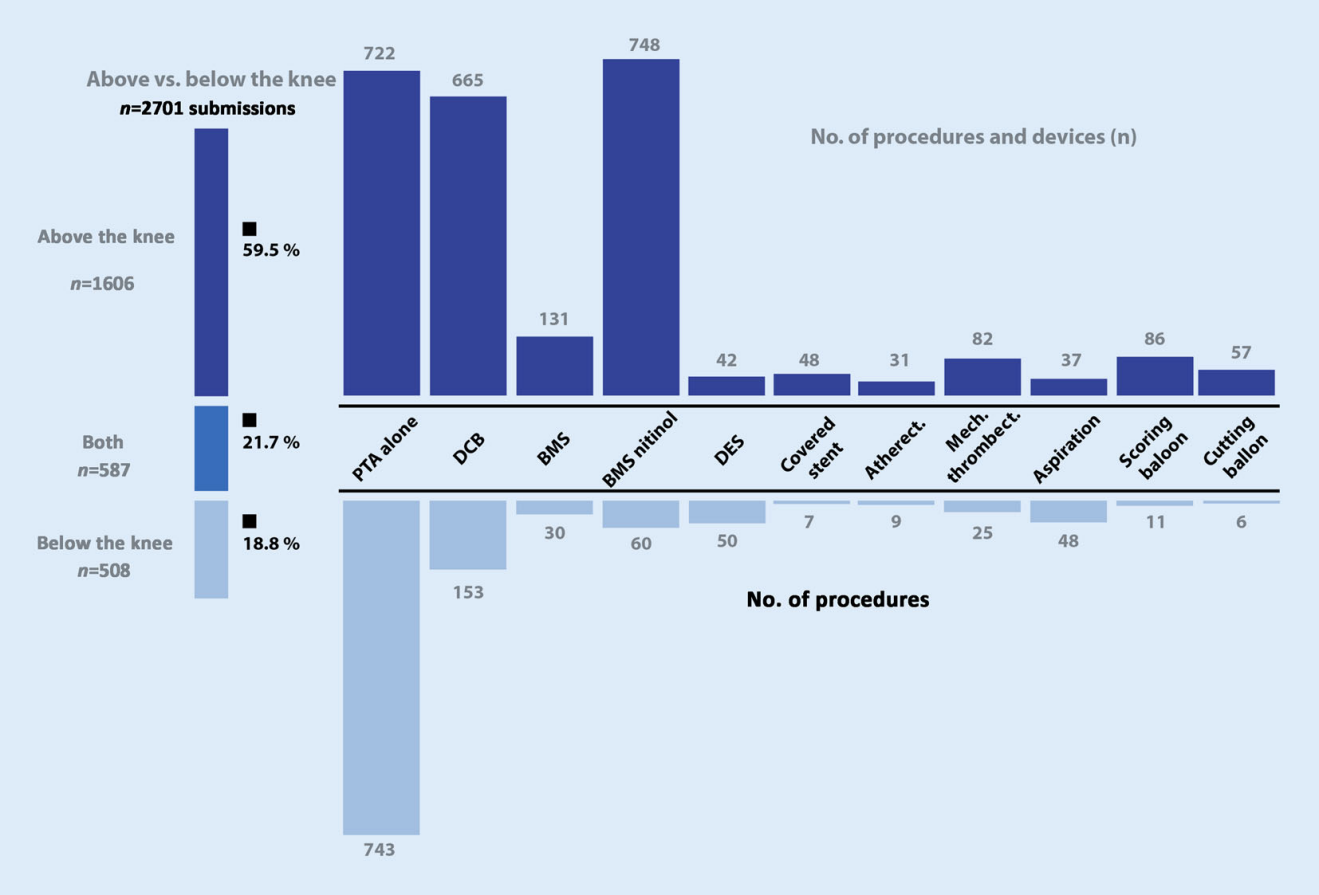
Distribution of the procedures and devices according to region (*PTA* percutaneous transluminal angioplasty, *DCB* drug-coated balloons, *Atherect.* atherectomy, *BMS* bare metal stents, *DES* drug-eluting stent, *mech. thrombect.* mechanical thrombectomy)

Data concerning the treated flow path were reported in 2701 cases (Fig. 3). The suprapopliteal flow path, down to and including the P2 segment was treated in 59.5 % of the patient cases, infrapopliteal vessels in 18.8 %. In 21.7 % of the cases, treatment of both sections was indicated. For stage II cases, the suprapopliteal and infrapopliteal flow areas were affected in 84.9 % and 4.9 %, respectively (10.3 % both). In patients suffering from CLI 38.7 % of the cases involved the suprapopliteal artery and 30.1 % the infrapopliteal (31.2 % both).Of the suprapopliteal interventions 722 were conducted using standard PTA (uncoated balloon PTA), while 665 employed drug-coated balloons and 748 nitinol bare metal stents. Only 48 of the implanted or employed suprapopliteal devices were covered stents. In the infrapopliteal segment (below P3) 743 procedures (i. e. the great majority) were performed exclusively using standard PTA. Only 153 of the devices used were coated balloons. Uncoated self-expanding stents were implanted in 60 cases.

### Anticoagulation medication

Information concerning anticoagulation therapy (both before and after interventions) can be found in Table 5. A distinction was made between the two antiplatelet drugs acetylsalicylic acid (ASA) and clopidogrel and specific anticoagulants. In addition to vitamin K antagonists (VKA, e. g. warfarin), so-called new oral anticoagulants (NOAC), e. g. Xarelto® (rivaroxaban) or Pradaxa® (dabigatran) were also registered. Of the patients 9.5 % (*n* = 267) were not taking any anticoagulation medication prior to the intervention, which rose to 11.3 % (*n* = 317) postintervention. Approximately 65.0 % of patients were taking ASA as home medication, while 3.3 % were under clopidogrel therapy. 10.3 % of patients received dual platelet inhibition with ASA and clopidogrel prior to treatment, increasing to 50.4 % following interventions (Table 5). Of the patients with atrial fibrillation 23.2 % (*n* = 104) had no a VKA, NOAC or heparin as part of the medication regime prior to treatment. Following treatment, 42.3 % received a VKA, while 22.9 and 16.5 % began NOAC or heparin therapy, respectively.

**Table 5:** Anticoagulation medication of treated patients prior to and following interventions (*italic* antiplatelet therapy)

*n* = 2798 Treatment cases	Medication	
	Prior to intervention	On discharge
No anticoagulation medication	267 (9.5 %)	317 (11.3 %)
No antiplatelet therapy	609 (21.8 %)	463 (16.5 %)
*ASA only (without clopidogrel)*	1818 (*65 %*)	743 (*26.6 %*)
*Clopidogrel only (without ASA)*	83 (*3.0 %*)	181 (*6.5 %*)
*ASA and clopidogrel (dual)*	288 (*10.3 %*)	1411 (*50.4 %*)
Vitamin K antagonists (e. g. warfarin)	289 (10.3 %)	216 (7.7 %)
New oral anticoagulants (NOAC, e. g. rivaroxaban)	162 (5.8 %)	153 (5.5 %)
Heparin (LMWH)	448 (16.0 %)	317 (11.3 %)
Other (e. g. abciximab)	34 (1.2 %)	32 (1.1 %)

*ASA* acetylsalicylic acid, *LMWH* low molecular weight heparin

### Intraoperative and postoperative complications

When evaluating the intraoperative and postoperative complications, a distinction was made between patients with IC and CLI. The groups did not differ in the number of intraoperative complications (Table 6) and postoperative complications as listed in Table 7 and 8. None of the patients suffering from IC died during the hospital stay, in contrast to 19 (1.3 %) patients with CLI. It was not possible to determine the stage of PAOD in any of the deceased patients; however, it was determined that such patients had significantly more risk factors than those who survived (Table 9). In particular, the proportions of atrial fibrillation (45.0 % vs. 16.0 %, *p* = 0.002), chronic kidney disease (35.0 % vs. 11.7 %, *p* = 0.006) and dialysis-dependency (10.0 % vs. 4.0 %, *p* = 0.190) were significantly higher in the deceased patient group.

**Table 6:** Distribution of complications during procedures according to treatment indications. Distribution according to treatment indication (intermittent claudication vs. critical limb ischemia)

	Intermittent claudication (*n* = 1259)	Critical limb ischemia (*n* = 1495)
Dissection	59/1259 (4.7 %)	45/1495 (3.0 %)
Embolism	23/1259 (1.8 %)	29/1495 (1.9 %)
Perforation	5/1259 (0.4 %)	10/1495 (0.7 %)
Other	11/1259 (0.9 %)	16/1495 (1.1 %)

**Table 7:** Distribution of severe complications following interventions according to treatment indications. Distribution according to treatment indication (intermittent claudication vs. critical limb ischemia)

	Intermittent claudication (*n* = 1259)	Critical limb ischemia (*n* = 1495)
MACE	1/1259 (0.1 %)	28/1495 (1.9 %)
Myocardial infarction	1/1259 (0.1 %)	8/1495 (0.5 %)
Stroke/TIA	0/1259 (0 %)	1/1495 (0.1 %)
Death (following discharge)	0/1259 (0 %)	19/1495 (1.3 %)

*MACE* major adverse cardiac events, *TIA* transient ischemic attack

**Table 8:** Distribution of other complications following interventions excluding major adverse cardiovascular events (MACE) according to treatment indications. Distribution according to treatment indication (intermittent claudication vs. critical limb ischemia)

	Intermittent claudication (*n* = 1259)	Critical limb ischemia (*n* = 1495)
Pulmonary complications	2/1259 (0.2 %)	17/1495 (1.1 %)
Pseudoaneurysm	14/1259 (1.1 %)	18/1495 (1.2 %)
Compartment syndrome	1/1259 (0.1 %)	4/1495 (0.3 %)
Reintervention (endovascular)	6/1259 (0.5 %)	16/1495 (1.1 %)
Reintervention (surgical)	4/1259 (0.3 %)	29/1495 (1.9 %)
Major amputation (unplanned)	0/1259 (0 %)	14/1495 (0.9 %)
Minor amputation (unplanned)	0/1259 (0 %)	13/1495 (0.9 %)
Hemorrhage (requiring revision)	10/1259 (0.8 %)	14/1495 (0.9 %)
Wound infections	1/1259 (0.1 %)	14/1495 (0.9 %)

**Table 9:** Risk factors and epidemiological data of fatalities in treatment cases compared with the total study population

	Fatalities	Total study population
	*n* = 20	*n* = 2798
Proportion of men/women (%)	14/6 (70/30 %)	1696/1065 (60.6/39.4 %)
Age (average, years median)	80.05 (79)	72.46 (74)
Proportion of primary interventions	12 (60.0 %)	1798/2704 (66.5 %)
Coronary heart disease	9 (45.0 %)	1025/2798 (36.6 %)
Acute coronary syndrome (in the previous 6 months)	1 (5.0 %)	67/2798 (2.4 %)
Atrial fibrillation	9 (45.0 %)	449/2798 (16.0 %)
Diabetes mellitus	11 (55.0 %)	1271/2798 (45.4 %)
Chronic renal disease (stage 4/GFR <30 ml/min)	7 (35.0 %)	327/2798 (11.7 %)
Dialysis dependency	2 (10.0 %)	112/2798 (4.0 %)
Stroke or TIA (in the previous 6 months)	0 (0 %)	34/2798 (1.2 %)

*GFR* glomerular filtration rate, *TIA* transient ischemic attack

### Length of hospital stay

**Fig. 4:**
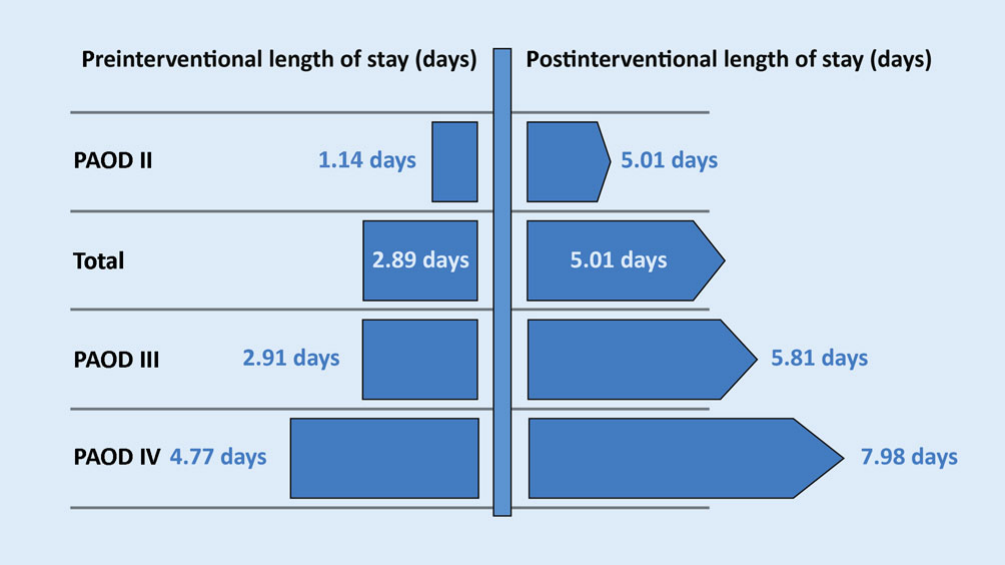
Length of hospital stay prior to and following interventions according to treatment indications

The length of stay (LOS) based on a total of 2696 valid data, was 7.8 days (median 4, minimum 0–maximum 280). Before intervention, the average LOS was 2.9 days (median 1, minimum 0–maximum 274) and postintervention 5.0 days (median 2, minimum 0–maximum 75). Average LOS for the individual stages of PAOD is presented in Fig. 4. It was found that the duration of hospitalization correlated with the severity or stage of PAOD. Patients with PAOD stage IV, for example, remained in hospital an average of 4.8 days (median 2, minimum 0–maximum 260) before intervention and a further 8.0 days (median 5, minimum 0–maximum 75) following treatment. Patients with PAOD stage II, however, only remained an average of 1.1 days (median 0, minimum 0–maximum 153) and 2.2 days (median 1, minimum 0–maximum 64) pretreatment and posttreatment, respectively.

120 (4.5 %) patients remained in hospital for less than 1 day, 90 (76.3 %) of which had IC and 28 (23.7 %) CLI. For the 2 remaining cases, staging was not possible. 482 (17.9 %) patients remained in hospital overnight, 7.3 % of patients suffering from and treated for PAOD stage II were discharged on the day of treatment, while 31.9 % remained overnight. The rates of discharge for patients treated for PAOD stages III and IV were 2.0 % and 5.4 %, respectively.

### Discharge destinations and results

Patency rate was used to gauge treatment result at the time of discharge, although no particular method for investigation was specified. It was shown that 58 (2.1 %) of the treated patients had closed reconstructions (1.9 % with IC, 2.4 % with CLI, 1.7 % following suprapopliteal reconstruction, 3.3 % following infrapopliteal reconstruction 3.3 % and 2.6 % following multiple level reconstruction). In 2432 (86.9 %) of the patients the reconstruction status was determined to be open or patent at the time of discharge. For the remaining 308 cases (11.0 %) the revascularization status could not be determined. On investigation of discharge destinations, a differentiation was made between transfer to another hospital, discharge to home or discharge to a nursing facility. Overall 83.8 % of the patients were able to return home following treatment, while 9.7 % (273) were either sent to a nursing home (5.3 %) or transferred to another hospital (4.4 %). A total of 20 patients (0.7 %) died during hospitalization. While 96.0 % of the patients with IC could be discharged to home only 74.1 % of patients suffering from CLI could return to private residences and 9.4 % of the latter group were transferred to a nursing facility (vs. 0.6 % for IC).

## Discussion

The goal of the present PSI study was to investigate the most common percutaneous endovascular practices and interventions for both suprapopliteal and infrapopliteal lesions in patients with IC and CLI, using data from the greatest possible number of patients from predominantly vascular surgery departments as well as cooperating radiology or angiology departments. Of the contacted 200 vascular centers, 79 (40 %) were willing to participate. In a consecutive 3‑month observation period, 2798 treatment cases could be included. This consecutive time frame was chosen in order to avoid any patient selection bias. Only the 74 centers that were able to ensure a continuous acquisition of patient data from the target population were included in the evaluation. The limited 3‑month survey period was also selected to minimize the amount of additional (non-remunerated) documentation work required from the participating hospitals. As it was not possible to monitor all of the trial centers, some of the information provided was incomplete and as a result the included population sizes differed somewhat between each variable. This aspect is indicated where relevant. The representativeness of the present study for the target population of all treated patients in Germany therefore remains to be determined, particularly as such a survey, as with all volunteer register surveys, depends on the number of centers willing to participate and take on the additional task of extra documentation. A limitation to the results of this study was due to the fact that primarily vascular surgery departments were involved. Furthermore, investigation of events following discharge (i. e. long-term clinical outcomes) were not the subject of the current investigation. The complications and events associated with the treatment described here are therefore limited to the period of hospitalization.

In 2009 Malyar et al. [4] described 50,180 endovascular revascularization procedures in patients in Germany with PAOD Fontaine stage IIB, 11,704 in patients with resting pain and 30,407 endovascular revascularizations in patients with CLI, all of which were inpatient procedures. Accordingly, endovascular interventions for patients suffering from IC represented more than half of all such treatment performed in Germany for this time period. In the patient population of the current study the proportion of patients with IC (45.7 %) was also very high, a fact which appears to conflict with guideline recommendations, which are more reserved when it comes to revascularization procedures of patients with IC. The German S3 guidelines state the following [1]: in patients suffering from intermittent claudication, supervised exercise programs aimed at increasing walking distance are similarly as effective as endovascular or vascular surgery interventions (recommendation grade A, evidence grade 1). In a systematic review, monitored walking exercise and PTA were also found to be equally effective [5]. Despite this, the use of monitored walking exercise as treatment has not been enforced and endovascular procedures remain the established practice for the treatment of IC. This was further demonstrated in the large nationwide inpatient sample (NIS) survey database (1999–2007) performed by Sachs et al. [6]. Here, 128,937 patients with IC received PTA +/- stent, while only 89,776 CLI patients received the same (all endovascular interventions including those which were suprainguinal); therefore, IC is not a less frequent indication for endovascular procedures compared to CLI.

In the PSI study patients suffering from CLI were both older and more severely ill than patients with IC. Diabetes mellitus, chronic kidney disease and coronary heart disease were all significantly more frequent in patients with CLI. Run-off was also significantly worse. Similar observations were also made by other authors [7, 8]. In the NIS (USA) database the proportion of diabetic patients with CLI was almost twice as high as those with IC, while the proportion of patients with reduced renal function was almost three times higher [9]. In accordance with the less favorable initial conditions, postoperative deaths were only documented for patients with CLI in the PSI study; however, the details of the postoperative complications should be interpreted with caution as should the low hospital mortality of 1.3 % in patients suffering from CLI. Due to organizational limitations in the PSI study patients were only observed up until the time of discharge from the hospital. Accordingly, hospital mortality was determined and not the standard 30-day mortality. Sachs et al. [6] were also only able to determine hospital mortality: 0.2 % of patients who received endovascular treatment for IC and 2.1 % of those who received the same for CLI. In the present study, the patency rate at discharge was also remarkably low. Only 2.1 % of patients had occluded revascularizations at the time of discharge. It must be noted, however, that in 11 % of cases patency of reconstruction was not documented at discharge; the results are therefore not valid and should be critically interpreted. In an earlier pilot study concerning endovascular treatment of IC and CLI, patency rates of 91.9 and 94.6 % were observed, respectively [10]. In the CRITISCH study, all forms of hemodynamic failure (including major amputations) following endovascular treatment were documented in patients with CLI and were observed at a total of 13 % [11].

It is important to stress that the present survey only secondarily serves as an outcome study in which the results of endovascular treatment of both IC and CLI were compared over a short time period. The primary objective was more to inventory current practices and techniques in the percutaneous endovascular therapy of infrainguinal PAOD in various departments, predominantly vascular surgery, as a pilot study (with the abovementioned restrictions). Data concerning this topic are completely lacking in Germany. Until this point, only estimated survey results have been published. For example, in a survey from Schmitz-Rixen et al. [12] 171 out of 223 (76.7 %) senior vascular surgeons described their departments as being a stand-alone department of vascular surgery, while 47 (21.1 %) were said to be a subdivision of general surgery and 5 (2.2 %) a subdivision of the cardiac surgery department. The proportion of radiology departments responsible for percutaneous arterial interventions was estimated to be 51–75 % from 16.7 % of survey respondents, to 76–99 % from 27.4 % and 100 % from 15.5 % of respondents whereas14.5 % reported that none of the interventions were performed as radiology procedures. Regarding vascular surgery, 8.3 % estimated the proportion of percutaneous arterial interventions to be 76–99 %, while 23.3 % estimated it to be 100 %. 8.2 % reported that, in the vascular surgery department, no percutaneous arterial interventions were carried out, while 40.9 % guessed the proportion of vascular surgery performing percutaneous interventions to be between 1 and 25 %. These estimations were further refined in the PSI study. In the vascular surgery departments, 60.1 % of percutaneous interventions were performed by vascular surgeons themselves, 32.8 % by radiologists and 6.9 % by angiologists. It was also possible to distinguish between individual preferences and habits. This concerned, among other things, the choice of inguinal approach/access, the majority of vascular surgeons preferred an inguinal antegrade approach. This technique is not often employed by angiologists; this group rather appears to prefer the inguinal retrograde crossover approach, which was selected in 61.1 % of cases. There were also significant differences in the choice of closure system, as shown in Table 4.

One point all guidelines agree on is postoperative antiplatelet therapy. In the German guidelines [1] the following is recommended: all patients should receive 100 mg ASA before, during and after interventions. Treatment should be continued for the long term, provided there are no contraindications (level of recommendation A, evidence level 1). Still under discussion is the option of a combination therapy with clopidogrel. In this case, the guidelines state more cautiously caution: following infrainguinal endovascular therapy with stenting, the temporary combination of ASA and clopidogrel can be recommended to improve the patency rate. Because the present study demonstrated that in a considerable proportion of cases (16.5 %) no postoperative antiplatelet therapy was documented, further clarification is urgently required in other cohort studies. Conversely, 5.5 % of patients were supplied with NOAC and 7.7 % with vitamin K antagonists, in apparent contrast to guideline recommendations (evidence level 1): oral anticoagulants are not to be used after PTA of femoropopliteal or tibial lesions; however, 8 % of patients with IC and 22.8 % of patients with CLI also suffered from atrial fibrillation. Whether this suffices as an indication for anticoagulation could not be determined, although it should be noted that relatively few patients with atrial fibrillation received treatment with NOACs or vitamin K antagonists.

From the NIS database, Sachs et al. [6] found the average length of hospital stay in patients receiving PTA +/- stent to be 1.0 +/- 0.02 days, although 87.8 % of the procedures were performed as same day surgery. Lo et al. [13] also used the NIS database. In their study, they reported over 230,469 endovascular procedures in patients with IC between 1998 and 2009. In 2009, 65 % of interventions in men and 61 % in women were performed as outpatient procedures. In CLI, however, they observed that inpatient endovascular procedures were performed 2.7 times more often than outpatient procedures. In comparison to these studies, the average length of hospital stay in the patient population of the present study is relatively long. Only 4.5 % of all treatments were carried out as outpatient or same day surgery procedures, although a distinct difference could be observed between patients with PAOD II and those with PAOD III/IV. For example, 7.3 % of patients treated for PAOD stage II were discharged on the day of treatment, while 31.9 % stayed overnight in hospital. In patients with PAOD III and IV the numbers were 2.0 and 5.4 %, respectively.

The analysis of the selected techniques used to treat suprapopliteal and infrapopliteal lesions proved to be an important point of investigation. Suprapopliteal lesions were most frequently treated with either PTA alone, a drug-coated balloon (DCB) or a self-expanding nitinol stent, all with relatively similar frequencies (Fig. 3). This distribution reflects the uncertainty in the various guidelines, recommendations of which range from the wide propagation of primary stent angioplasty with nitinol stents, to preference of balloon angioplasty with secondary stenting (bail out) [14]. At the present time a general recommendation does not exist. This was also demonstrated in a Cochrane review by Chowdhury et al. [15]. These authors addressed the question of whether implantation of uncoated metal stents improved the vessel patency in symptomatic PAOD patients with lesions of the superficial femoral artery, in comparison to PTA. The analysis of a total of 11 studies (1387 patients) demonstrated a significant improvement in patency rate (determined by angiography) at 6 months following intervention for PTA + stent versus PTA alone (odds ratio 2.9); however, this advantage could not longer be observed after 12 months. Following this time, no differences in walking distance (measured on a treadmill) or ankle brachial index (ABI) could be measured. The same was true for the quality of life. The data support the recommendations of the National Institute for Health and Care Excellence (NICE) [16], which advise physicians to exercise restraint in the placement of stents. Jens et al. [17] came to a similar conclusion in their systematic review and meta-analysis, where they name PTA as the method of choice for above the knee endovascular interventions in patients with IC, with optional stenting (bail out) in cases of inadequate balloon angioplasty. The latest systematic review presented yet more new recommendations to the National Health Service (NHS) of the UK, this time from an economic standpoint [18]. According to this analysis, drug-coated balloons (DCB) and drug-eluting stents (DES) provide the greatest clinical and economic benefits in the endovascular treatment of suprapopliteal lesions compared to PTA with uncoated balloons and/or PTA with bare metal stents (BMS). In infrapopliteal lesions, the varieties of endovascular techniques used in the PSI study were of minor importance: PTA alone was predominantly favored (*n* = 743), with DCB being implemented in significantly fewer cases (*n* = 153). This is in agreement with the current guideline recommendations. Whether this approach will endure, amongst numerous other technical possibilities, is yet to be determined.

The original aim of the PSI study was to use data to describe the current or initial situation, which would serve as a baseline and on future reinvestigation allow researchers to observe trends in treatment strategies. Furthermore, it will be possible to determine how quickly results of (randomized) studies can and will be implemented in actual clinical practice, also from an economic perspective. The vast multitude of devices and procedures employable for such treatments, even when some are only rarely implemented (Fig. 3), highlights the need for further clarification.

We would like to express our sincere gratitude for the encouraging commitment of the participating centers in the documentation and reporting of data from all cases. In 2 years time we plan on revisiting this investigation in order to further discuss and review the trends discussed in this article.

## Conclusions for practice

- In the PSI study 60.1 % of the percutaneous endovascular procedures in vascular centers run by a vascular surgeon were performed by vascular surgeons themselves, 32.8 % by radiologists and 6.9 % by angiologists. This emphasizes the importance of cooperation in the treatment of vascular patients.
- Patients with intermittent claudication and critical limb ischemia significantly differed in terms of risk factors, particularly with respect to chronic renal disease, diabetes mellitus and cardiac risk factors. Of the patients categorized as PAOD stage II, 41.3 % presented with 3 patent crural vessels compared with only 10.8 % of patients in stage IV.
- For suprapopliteal lesions, self-expanding bare-metal stents (nitinol) were used in preference to PTA with uncoated and DCB. Standard PTA, on the other hand, was the most popular technique for infrapopliteal lesions.
- With respect to serious complications that can arise during intervention, percutaneous endovascular treatment of intermittent claudication is considered to be a safe procedure (serious complications in less than 1 % and no deaths).
- Only 4.5 % of all treatments were performed under outpatient/day surgery conditions. Of patients with PAOD II, however, only 31.9 % remained in hospital overnight.

## Caption Electronic Supplementary Material




