# Supplementary material for: Percutaneous endovascular treatment of infrainguinal PAOD: Results of the PSI register study in 74 German vascular centers
Source: Gefasschirurgie. 2016 Oct 28;22(Suppl 1):17–27. doi: 10.1007/s00772-016-0202-2 (PMC5306226; doi:10.1007/s00772-016-0202-2)
Supplement: Supplementary file 1 [file 772_2016_202_MOESM1_ESM.pdf]

Elmshorn	Klinik für Allgemein-, Viszeral- und Gefäßchirurgie<br>Zertifiziertes Gefäßzentrum (DGG, DGA) Elmshorn<br>Regio Klinik Elmshorn	Herr	Dr. med.	Christos Petridis
Elmshorn	Klinik für Allgemein-, Viszeral- und Gefäßchirurgie<br>Zertifiziertes Gefäßzentrum (DGG, DGA) Elmshorn<br>Regio Klinik Elmshorn	Herr	Dr. med.	Oliver Hader
München	Helios Klinikum München West<br>Klinik für Gefäßchirurgie<br>Zentrum für vaskuläre und endovaskuläre Chirurgie	Herr	Dr. med.	Reza Ghotbi
Kassel	Agaplesion Diakonie Kliniken Kassel<br>Abteilung für Gefäßchirurgie	Herr	Prof. Dr. med.	Thomas Bürger
Bautzen	Oberlausitz-Kliniken gGmbH<br>Krankenhaus Bautzen<br>Chirurgische Klinik, Gefäßchirurgische Abteilung	Herr	Dr. med.	Herold Kuffner
Bautzen	Oberlausitz-Kliniken gGmbH<br>Krankenhaus Bautzen<br>Chirurgische Klinik, Gefäßchirurgische Abteilung / Asklepios Birkenwerder	Herr		Eckhard Stautner
Bonn	Abteilung für Gefäßchirurgie<br>Gemeinschaftskrankenhaus Bonn<br>St. Elisabeth - St. Petrus - St. Johannes gGmbH	Herr	Dr. med.	Jürgen Remig
Lingen	St. Bonifatius Hospital Lingen<br>Chirurgische Klinik<br>Fachbereich Gefäßchirurgie	Herr	Dr. med.	Jörg Teßarek
Lingen	St. Bonifatius Hospital Lingen<br>Chirurgische Klinik<br>Fachbereich Gefäßchirurgie	Herr	Dr. med.	Hartmut Görtz
Hamburg	Medizinisches Versorgungszentrum<br>Prof. Mathey, Prof. Schofer GmbH	Herr	PD Dr. med.	Sebastian Sixt

Hamburg	Medizinisches Versorgungszentrum<br>Prof. Mathey, Prof. Schofer GmbH<br>Medical Care Center	Herr	Prof. Dr. med.	Joachim Schofer
Reinbek	Krankenhaus Reinbek St. Adolf-Stift<br>Abteilung für Diagnostische und Interventionelle Radiologie	Herr	Prof. Dr. med.	Gerrit Krupski-Berdien
Reinbek	Krankenhaus Reinbek St. Adolf-Stift<br>Chirurgische Klinik<br>Abteilung für Gefäßchirurgie	Herr	Dr. med.	Matthias Schneider
Chemnitz	Klinikum Chemnitz gGmbH, Standort Küchwald<br>Klinik für Thorax-, Gefäß- und endovaskuläre Chirurgie	Herr	Dr. med.	Sven Seifert
Landshut	Krankenhaus Landshut-Achdorf<br>Chirurgische Klinik III<br>Abteilung Gefäßchirurgie	Herr	Dr. med.	Johann Hatzl
Birkenwerder	Asklepios Klinik Birkenwerder<br>Klinik für Gefäßchirurgie und Endovaskuläre Chirurgie	Herr	Dr. med.	Mario Kuhnert
Vogtareuth	Schön Klinik Vogtareuth<br>Klinik für operative und interventionelle Gefäßchirurgie	Herr	Dr. med.	Marcel Hofmann
Koblenz	Gemeinschaftsklinikum Mittelrhein gGmbH<br>Zentrum für Gefäßmedizin und Wundbehandlung	Herr	PD Dr. med.	Gunnar Riepe
Bonn	GFO Kliniken Bonn, Betriebsstätte St. Marien<br>Abteilung für Gefäß- und Endovaskularchirurgie	Herr	Prof. Dr. med.	Kai M. Balzer
Flensburg	Ev.-Luth. Diakonissenanstalt zu Flensburg<br>Gefäßchirurgische Klinik	Herr	Dr. med.	Knut P. Walluscheck

Saalfeld	Thüringen-Kliniken<br>"Georgius Agricola" GmbH<br>Klinik für Gefäßchirurgie -<br>Zentrum für Gefäßmedizin	Herr	Dr. med.	Thomas Krönert
Göppingen	Alb Fils Kliniken<br>Klinik am Eichert<br>Allgemeinchirurgische Klinik -<br>Schwerpunkt Gefäßchirurgie	Herr	Dr. med.	Peter Richter
Mühlheim a. d. R.	Evangelisches Krankenhaus<br>Mülheim<br>Gefäßchirurgische Klinik	Herr	PD Dr. med.	Alexander Stehr
Celle	Allgemeines Krankenhaus Celle<br>Klinik für Gefäßchirurgie	Herr	Dr. med.	Uwe Brune
Hamburg	Agaplesion Diakonieklinikum<br>Hamburg gGmbH<br>Klinik für Gefäßmedizin	Herr	Dr. med.	Wolfgang Paul Tigges
Bielefeld	Evangelisches Krankenhaus<br>Bielefeld<br>Gefäßklinik-Johannesstift	Herr	Dr. med.	Burkhard Feidicker
Bielefeld	Evangelisches Krankenhaus<br>Bielefeld<br>Gefäßklinik-Johannesstift	Herr	Dr. med.	Ulrich Quellmalz
Oldenburg	Pius-Hospital Oldenburg<br>Medizinischer Campus<br>Universität Oldenburg<br>Klinik für Thorax-, Gefäß- und<br>endovaskuläre Chirurgie /<br>Oldenburger Gefäßzentrum	Herr	Dr. med.	Andreas Cöster
Hamburg	Kardiologie am Tibarg &<br>Hoheluft<br>Hamburg	Herr	Prof. Dr. med.	Dirk Walter
Speyer	Diakonissen-Stiftungs-<br>Krankenhaus Speyer<br>Klinik für Gefäßchirurgie	Herr	Prof. Dr. med	Gerhard Rümenapf
Köln	Uniklinik Köln<br>Herzzentrum / Klinik und<br>Poliklinik für Gefäßchirurgie	Herr	Prof. Dr. med.	Jan Brunkwall

Bad Nauheim	Kerckhoff-Klinik Bad Nauheim<br>Klinik für Gefäßchirurgie / Harvey Gefäßzentrum	Herr	Dr. med.	Simon Classen
Traunstein	Klinikum Traunstein<br>Gefäßzentrum Ostbayern	Herr	Dr. med.	Volker Kiechle
Soest	Klinikum Stadt Soest<br>Klinik für Gefäß- und endovaskuläre Chirurgie / Westfälisches Herz- und Gefäßzentrum	Herr	Dr. med.	Kristian Nitschmann
Hamburg	Universitäres Herzzentrum Hamburg GmbH<br>Klinik und Poliklinik für Gefäßmedizin / Gefäßchirurgie - Angiologie - Endovaskuläre Therapie	Herr	Prof. Dr. med.	Eike Sebastian Debus
Trier	Krankenhaus der Barmherzigen Brüder Trier<br>Abteilung für Allgemein-, Viszeral- und Gefäßchirurgie / Zentrum für Gefäßmedizin	Herr	Prof. Dr. med.	Detlef M. Ockert
Lübeck	Universitätsklinikum Schleswig-Holstein<br>Campus Lübeck<br>Klinik für allgemeine Chirurgie<br>Bereich Gefäß- und endovasculäre Chriurgie	Herr	Prof. Dr. med.	Markus Kleemann
Lübeck	Universitätsklinikum Schleswig-Holstein<br>Campus Lübeck<br>Klinik für allgemeine Chirurgie<br>Bereich Gefäß- und endovasculäre Chriurgie	Herr	Dr. med.	Marcus Wiedner
Gera	SRH Wald-Klinikum Gera<br>Thorax- und Gefäßchirurgie/Angiologie<br>Gefäßzentrum?	Herr	PD Dr. med.	Thomas Lesser
Suhl	SRH Zentralklinikum Suhl<br>Klinik für Gefäß- und Thoraxchirurgie<br>Herz-Lungen-Gefäßzentrum	Herr	Dr. med.	Klaus-Dieter Thom

München	Städtisches Klinikum München<br>Klinikum Neuperlach<br>Klinik für Gefäßchirurgie, vaskuläre und endovaskuläre Chirurgie	Herr	PD Dr. med.	Rolf Weidenhagen
Nordhausen	Südharz Klinikum Nordhausen<br>Klinik für Gefäßchirurgie, vaskuläre und endovaskuläre Chirurgie	Herr	Dr. med.	Wieland Gunkel
Garmisch-Patenkirchen	Klinikum Garmisch-Partenkirchen<br>Abteilung für Gefäßchirurgie	Herr	Dr. med.	Stefan Nöldeke
Garmisch-Patenkirchen	Klinikum Garmisch-Partenkirchen<br>Abteilung für Gefäßchirurgie	Herr	Dr. med.	Thomas Lange
Essen	Elisabeth-Krankenhaus Essen<br>Klinik für Gefäßchirurgie und Phlebologie / Contilia Herz- und Gefäßzentrum	Herr	Prof. Dr. med.	Johannes Hoffmann
Viechtach	Arberland Kliniken<br>Kreiskrankenhaus Viechtach<br>Zentrum für Gefäßmedizin und Gefäßchirurgie	Herr	Dr. med.	Julio Perez Delgado
Paderborn	St. Vincenz-Krankenhaus Paderborn<br>Klinik für Gefäßchirurgie	Herr	Dr. med.	Jörg Forkel
Senftenberg	Klinikum Niederlausitz<br>Gefäßzentrum Niederlausitz<br>Klinik für Gefäßchirurgie	Herr		Tom Hammermüller
Senftenberg	Klinikum Niederlausitz<br>Gefäßzentrum Niederlausitz<br>Klinik für Gefäßchirurgie	Herr	Dr. med.	Frank Thomas Wittstock
Mühlhausen	Hufeland Klinikum<br>Standort Mühlhausen<br>Klinik für Chirurgie - Abteilung für Gefäßchirurgie<br>Hufeland Gefäßzentrum	Herr		Jan Gräbedünkel
Wittlich	Verbundkrankenhaus Bernkastel / Wittlich<br>Gefäßzentrum Wittlich	Frau	Dr. med.	Erica Blajan

Wittlich	Verbundkrankenhaus Bernkastel / Wittlich<br>Gefäßzentrum Wittlich	Herr	Dr. med.	Dirk Lommel
Karlsruhe	Städtisches Klinikum Karlsruhe<br>Klinik für Gefäß- und Thoraxchirurgie<br>Gefäßzentrum auch?	Herr	Prof. Dr. med.	Martin Storck
Karlsruhe	Städtisches Klinikum Karlsruhe<br>Klinik für Gefäß- und Thoraxchirurgie<br>Gefäßzentrum auch?	Herr	Prof. Dr. med.	Peter Reimer
Villingen-Schwenningen	Schwarzwald-Baar Klinikum Villingen-Schwenningen<br>Klinik für Gefäßchirurgie und Gefäßmedizin / Gefäßzentrum	Herr	Dr. med.	Stephan Eder
Neustadt	Krankenhaus Hetzelstift Neustadt/Weinstraße<br>Klinik für operative und enovaskuläre Gefäßchirurgie	Herr	Dr. med.	Mathias Wenk
Bad Wildungen	Asklepios Stadtklinik Bad Wildungen<br>Klinik für Gefäßchirurgie und endovaskuläre Gefäßtherapie	Herr	Dr. med.	Peter Dahl
Rendsburg	imland Klinik Rendsburg<br>Gefäß- und Thoraxchirurgie	Herr	Dr. med.	Markus Siggelkow
Saarbrücken	Caritasklinikum Saarbrücken St. Theresia<br>Klinik für Gefäß- und Endovascularchirurgie	Frau	Dr. med.	Ulrike Ossig
Ludwigsburg	Klinikum Ludwigsburg<br>Klinik für Gefäßchirurgie, Vaskuläre und endovaskuläre Chirurgie	Herr	PD Dr. med.	Johannes Gahlen

Hamburg	Cardiologicum Hamburg	Herr	Dr. med.	Georg Schmidt
Saarbrücken	CaritasKlinikum Saarbrücken St. Theresia<br>Medizinische Klinik - Interventionelle Kardiologie und Angiologie	Herr	Prof. Dr. med	Michael Kindermann
Saarbrücken	CaritasKlinikum Saarbrücken St. Theresia<br>Medizinische Klinik - Interventionelle Kardiologie und Angiologie	Herr	PD Dr. med	Magnus Baumhäkel
Düsseldorf	Universitätsklinikum Düsseldorf<br>Klinik für Gefäß- und Endovaskulärchirurgie	Herr	Prof. Dr. med.	Hubert Schelzig
Düsseldorf	Universitätsklinikum Düsseldorf<br>Klinik für Gefäß- und Endovaskulärchirurgie	Herr	PD Dr. med.	Alexander Oberhuber
Bremen	Klinikverband Gesundheit Nord<br>Klinikum Bremen-Mitte<br>Klinik für Gefäßchirurgie	Herr	PD Dr. med.	Letterio Barbera
Bernburg	Ameos Klinikum Bernburg<br>Klinik für Allgemein-, Visceral-, Gefäß- und endovaskuläre Chirurgie, Phlebologie	Herr	Dr. med.	Klaus-Dieter Wagenbreth
München	Klinikum der Universität München<br>Campus Innenstadt<br>Sektion Gefäßchirurgie	Herr	Dr. med.	Ramin Banafsche
Moers	Stiftung Krankenhaus Bethanien<br>Klinik für Gefäßchirurgie	Herr	Prof. Dr. med.	Bruno Geier

Nürnberg	Klinik Martha Maria<br>Klinik für Allgemein-, Visceral- und Gefäßchirurgie<br>Abteilung Gefäßchirurgie	Herr	Dr. med.	Thomas Noppeney
Hamburg	Evangelisches Amalie Sieveking-Krankenhaus<br>Klinik für Gefäß- und endovaskuläre Chirurgie	Herr	Dr. med.	Peter Breuer
Hamburg	Evangelisches Amalie Sieveking-Krankenhaus<br>Klinik für Gefäß- und endovaskuläre Chirurgie	Herr	Dr. med.	Axel Pflugradt
Haan	St. Josef Krankenhaus Haan<br>Abteilung für Gefäßchirurgie/Gefäßzentrum Kplus	Herr	Dr. med.	Udo Huberts
Berlin	HELIOS Privatklinik Berlin-Zehlendorf<br>Gefäßzentrum Berlin Südwest<br>Klinik für arterielle und endovaskuläre Gefäßchirurgie	Herr	Dr. med.	Michael Naundorf
Hamburg	Herz- und Gefäßmedizin Esplanade<br>Kardiologisch-Angiologische Praxis	Herr	Dr. med.	Harald Dill
Aachen	Uniklinik RWTH Aachen<br>Klinik für Gefäßchirurgie	Herr	Prof. Dr. med.	Michael Jacobs
Lünen	St.-Marien Hospital Klinikum Lünen<br>Gefäßchirurgische Klinik	Herr	Dr. med.	Thomas Fährenkemper
Bremen	Klinikverband Gesundheit Nord<br>Klinikum Bremen-Nord<br>Klinik für Allgemein-, Gefäß- und Visceralchirurgie	Herr	Prof. Dr. med.	Heiner Wenk

Hannover	Medizinische Hochschule Hannover<br>Klinik für Herz-, Thorax-, Transplantations- und Gefäßchirurgie	Herr	Prof. Dr. med.	Omke Enno Teebken
Unna	Evangelisches Krankenhaus Unna<br>Klinik für Gefäßchirurgie	Herr	Dr. med.	Johannes Wilde
Bad Salzungen	Klinikum Bad Salzungen<br>Klinik für Gefäßchirurgie	Herr	Dipl.-Med.	Thomas Schaefer
Esslingen	Klinikum Esslingen<br>Klinik für Gefäß- und Thoraxchirurgie	Herr	Prof. Dr. med.	Florian Liewald
Meppen	Krankenhaus Ludmillenstift<br>Abteilung für Allgemein-, Visceral- und Gefäßchirurgie	Herr	Dr. med.	Volker K. Lauff
Görlitz	Städtisches Klinikum Görlitz<br>Klinik für Allgemein-, Viszeral- und Gefäßchirurgie	Herr	Dr. med.	Marco Krahl
Hannover	KRH Klinikum Region Hannover<br>KRH Klinikum Robert Koch Gehrden<br>Interdisziplinäres Gefäßzentrum	Herr	Dr. med.	Götz Voshage
Hannover	KRH Klinikum Region Hannover<br>KRH Klinikum Robert Koch Gehrden<br>Interdisziplinäres Gefäßzentrum	Herr		Przemyslaw Kolka
Ulm	Universitätsklinikum Ulm<br>Zentrum für Chirurgie<br>Klinik für Herz-, Thorax- und Gefäßchirurgie<br>Sektion Gefäßchirurgie	Herr	Prof. Dr. med.	Karl-Heinz Orend

Mönchengladbach	Kliniken Maria Hilf Mönchengladbach<br>Klinik für Gefäßchirurgie und Angiologie	Frau	Prof. Dr. med	Barbara Weis-Müller
Dortmund	Katholische St. Lukas Gesellschaft<br>St. Rochus-Hospital Castrop-Rauxel<br>Katholisches Krankenhaus Dortmund West<br>Klinik für Chirurgie	Herr	Dr. med.	Hinrich Böhner
